# Pediatric Parapneumonic Empyema, Spain

**DOI:** 10.3201/eid1409.071094

**Published:** 2008-09

**Authors:** Ignacio Obando, Carmen Muñoz-Almagro, Luis A. Arroyo, David Tarrago, David Sanchez-Tatay, David Moreno-Perez, Sahar S. Dhillon, Cristina Esteva, Susanna Hernandez-Bou, Juan J. Garcia-Garcia, William P. Hausdorff, Angela B. Brueggemann

**Affiliations:** Virgen del Rocio Children’s Hospital, Seville, Spain (I. Obando, L.A. Arroyo, D. Sanchez-Tatay); Sant Joan de Deu Hospital, Barcelona, Spain (C. Muñoz-Almagro, C. Esteva, S. Hernandez-Bou, J.J. Garcia-Garcia); Spanish Reference Laboratory for Pneumococci, Madrid, Spain (D. Tarrago); Carlos de Haya Children’s Hospital, Malaga, Spain (D. Moreno-Perez); University of Oxford, Oxford, UK (S.S. Dhillon, A.B. Brueggemann);; GlaxoSmithKline Biologicals, Rixensart, Belgium (W.P. Hausdorff)

**Keywords:** *Streptococcus pneumoniae*, pediatric parapneumonic empyema, serotype 1, epidemiology, research

## Abstract

Pediatric parapneumonic empyema (PPE) has been increasing in several countries including Spain. *Streptococcus pneumoniae* is a major PPE pathogen; however, antimicrobial pretreatment before pleural fluid (PF) sampling frequently results in negative diagnostic cultures, thus greatly underestimating the contribution of pneumococci, especially pneumococci susceptible to antimicrobial agents, to PPE. The study aim was to identify the serotypes and genotypes that cause PPE by using molecular diagnostics and relate these data to disease incidence and severity. A total of 208 children with PPE were prospectively enrolled; blood and PF samples were collected. Pneumococci were detected in 79% of culture-positive and 84% of culture-negative samples. All pneumococci were genotyped by multilocus sequence typing. Serotypes were determined for 111 PPE cases; 48% were serotype 1, of 3 major genotypes previously circulating in Spain. Variance in patient complication rates was statistically significant by serotype. The recent PPE increase is principally due to nonvaccine serotypes, especially the highly invasive serotype 1.

## Abstract 2

Increased incidence is principally due to highly invasive nonvaccine serotypes of pneumococci, especially serotype 1.

Pleural effusions occur in at least 40% of children hospitalized with bacterial pneumonia. Occasionally, the infectious agent invades the pleura to cause pediatric paraneumonic empyema (PPE) (*1*), characterized by the presence of pus. Although rarely associated with fatalities in industrialized countries, PPE often results in prolonged hospitalization and surgical intervention, and patients are at risk for serious and long-lasting illness (*2*,*3*).

An increasing incidence of PPE has been reported in several countries since the mid-1990s (*2*–*6*), but it is not clear why. *Streptococcus pneumoniae* is the most frequently found microorganism in most recent reports. However, conventional microbiologic culture methods have low sensitivity, usually because of antimicrobial pretreatment before sterile-site sampling. Consequently, the contribution of antimicrobial drug–susceptible serotypes might be higher than reported estimates. Molecular and antigen detection-based techniques, including direct molecular typing of culture-negative pleural fluid (PF) samples (*7*), can be useful adjuncts in defining the contributory role of different microorganisms and pneumococcal serotypes to PPE etiology (*4*,*8*).

Our study’s goal was to prospectively investigate the molecular epidemiology of pneumococcal PPE among children admitted to 3 of the largest tertiary-care pediatric hospitals in Spain. There were 4 objectives: 1) identify the serotypes and multilocus sequence typing (MLST) genotypes causing PPE and determine whether a temporal change in the circulating genotypes could explain the recent increase; 2) determine whether the causal genotypes were only associated with PPE or also caused other invasive pneumococcal disease (IPD) in the same population, or were carried by healthy children; 3) compare serotypes and genotypes recovered from northern and southern Spain in the context of regional differences in 7-valent pneumococcal conjugate vaccine (PCV7) uptake; and 4) identify any differences between highly invasive serotypes and more opportunistic serotypes with respect to epidemiology and inflammatory markers.

## Methods

### Prospective and Retrospective Identification of PPE Cases

PPE cases involving all children <18 years of age at Sant Joan de Deu Hospital Barcelona, Spain, were prospectively enrolled beginning October 1, 2003. PPE patients <14 years of age admitted to Virgen del Rocío Children’s Hospital (VRCH) in Seville and Carlos de Haya Children’s Hospital (CHCH) in Malaga were prospectively enrolled beginning January 1, 2005. The study period extended to June 30, 2006, in all locations for molecular analyses and through December 2006 for trend analyses. Eligible patients were identified through notification by attending physicians or by clinical microbiology laboratories when a sterile site sample was pneumococcal culture or pneumolysin (*ply*) positive. Participating centers served a pediatric referral population of 607,796 (9% of the corresponding Spanish population).

PPE was defined according to published criteria (*6*). Requirements for thoracocentesis, decortication, administration of fibrinolytics, and antimicrobial drug therapy were determined according to usual clinical practice. Patients with PPE not requiring thoracocentesis or surgical decortication were excluded, as were cases with PF analysis consistent with a transudate. Thoracocentesis was performed by pediatric surgeons, except for acutely ill patients who were tapped in the emergency department or intensive-care unit. PF specimens were sent for routine microbiologic culture and biochemical analysis; remaining fluid was frozen at –80ºC for further molecular testing. Clinical, demographic, and outcome data were collected by using a standardized case report form.

To identify temporal trends, we also retrospectively identified PPE cases at VRCH and CHCH during 1998–2004 using International Classification of Diseases, 9th Revision, codes for empyema (510.0 or 510.9) and chart review. PPE definition and patient exclusion criteria were the same as those used in the prospective study.

### Nasopharyngeal Carriage Study

From January 2005 through June 2006, nasopharyngeal (NP) swab specimens were obtained from 635 children 6 months to 6 years of age attending 4 primary healthcare centers for well-child visits and 2 hospital emergency rooms in Seville. Exclusion criteria were chronic medical condition, moderate to severe acute process including fever >39ºC, lower respiratory tract infection, vomiting, dehydration, or other ill appearance. The study was conducted according to World Health Organization recommendations (*9*)

### Informed Consent

Written informed consent was obtained from parents or legal guardians of participating children before thoracocentesis or nasopharyngeal swabbing. Hospital ethics committees approved the studies.

## Retrospective Analysis of IPD Cases

IPD cases from patients at VRCH and CHCH during 2001–2006 were retrospectively ascertained from microbiology department databases of both centers and confirmed by chart review. Viable pneumococcal isolates were serotyped (70% of cases) by the Spanish Reference Laboratory of Pneumococci and genotyped by MLST (61% of cases).

### Testing of PF Samples

Pneumococci were identified by using microbiologic and molecular genotyping methods; susceptibility testing was performed by agar dilution (*10*), and Clinical Laboratory Standards Institute interpretive criteria were used to define susceptibility (*10*). Culture-negative PFs were assayed for the presence of the pneumolysin (*ply*) gene, by using a real-time PCR in Barcelona adapted from Corless et al. (*11*) and a published assay (*12*) in Seville. Conventional serotyping using the Quellung method was performed where possible; culture-negative or incompletely genotyped PFs were serotyped by using a real-time PCR that targets different capsular locus genes (*13*). DNA was extracted from PFs by using 20% wt/vol Chelex-100 resin (Bio-Rad Laboratories, Hercules, CA, USA) in Barcelona and the Nucleospin kit (Clontech Laboratories, Inc., Mountain View, CA, USA) in Seville.

### Molecular Genotyping

MLST was performed by using standard methods (*14*), with the exception of a change in PCR primers for the *gdh*, *recP*, *xpt* genes when genotyping PFs directly; in the PCR amplification step, first-round primers from a nested PCR (*15*) were substituted for standard MLST primers to increase sensitivity. Allele and sequence type (ST) designations were made by using the MLST website (www.mlst.net).

### Statistical Analyses

Statistical analyses were performed by using SPSS for Windows version 14.0 (SPSS, Inc., Chicago, IL, USA). Reported p values were 2-tailed, and the level of significance was set at 0.05. Analysis of categorical variables was performed with the χ^2^ test and Fisher exact test, as appropriate. Continuous variables were compared by analysis of variance followed by a Bonferroni test for multiple comparisons. When data were not normally distributed, we used the Kruskal-Wallis test and conducted posterior comparison between individual groups using the Mann-Whitney U test with the Bonferroni correction. IPD potential was estimated by using a standard odds ratio and Mantel-Haenszel confidence intervals (*16*).

## Results

### Overall PPE Trends

**Figure 1:**
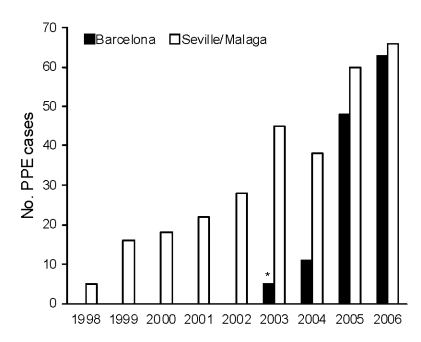
Annual number of pediatric parapneumonic empyema (PPE) cases among children <14 years of age admitted to Seville and Malaga hospitals from 1998 to June 2006 (combined prospective and retrospective data) and among children <18 years of age admitted to a Barcelona hospital from October 2003 through June 2006. *October 1, 2003, through December 31, 2003.

In Seville and Malaga, the annual number of PPE cases increased 13-fold (5 to 66 cases) during 1998–2006 (Figure 1). In Barcelona, the annual number of PPE cases increased from 11 cases in 2004 to 62 cases in 2006 (data before October 2003 were not available). Over these study periods, no obvious changes in referral patterns, overall pediatric population, guidelines for evaluating children with fever, pneumonia or PPE, or recommendations for performing diagnostic thorachocentesis in children with PPE were found. Table 1 describes the demographic characteristics of the 208 PPE patients prospectively enrolled during the molecular analysis study period (n = 98, Seville and Malaga; n = 110, Barcelona). There were no deaths.

**Table 1:** Demographic characteristics of 208 patients with PPE enrolled during the molecular analysis study period*

Characteristic	Value
Age, mo, mean ± SD (range)	51.8 ± 31 (2–180)
Gender ratio, M/F	1.06
Underlying disease, %†	4
Oral antimicrobial drugs before admission, %‡	29
Antimicrobial drug free before thoracocentesis, %§	23
PCV7 >1 dose, %	31
Referral, %	38

*PPE, pediatric parapneumonic empyema; PCV7, 7-valent pneumococcal conjugate vaccine. †Underlying disease included (no. patients): lymphoma (2), congenital heart disease (2), mild psychomotor retardation (2), varicella zoster infection (2) and genetic disease (1). ‡Median duration: 3 d, range 1–17 d. §100/147 children who had not been treated with oral antimicrobial drug therapy before admission received intravenous antimicrobial drug treatment before thoracocentesis for a median of 2 d (range 1–10 d).

### Microbiologic Evaluation

**Figure 2:**
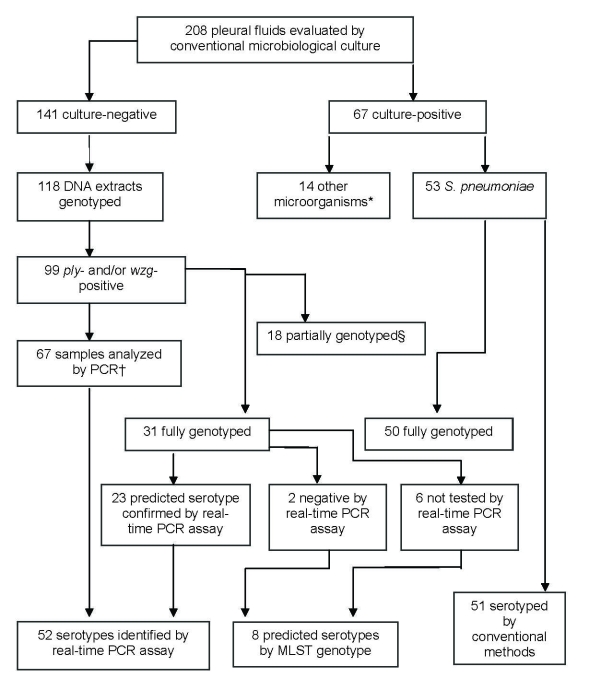
Microbiologic characteristics of pleural fluid (PF) specimens from pediatric parapneumonic empyema (PPE) case-patients. *Streptococcus pyogenes (6), *Staphylococcus aureus* (3), *Mycobacterium tuberculosis* (2), *Escherichia coli* (1)*, Streptococcus mitis (1), Peptostreptococcus* spp. (1). †Pleural fluids analyzed by PCR included 2 samples that were *ply* negative but *wzg* positive. ‡18 partially genotyped by multilocus sequence typing (MLST) (>3 alleles), as DNA concentration was too low for reliable PCR amplification and sequencing.

Sixty-seven (32%) patients had positive blood and/or PF cultures for any pathogen, and *S. pneumoniae* was isolated from 53 (79%) of these cases (Figure 2). In 51 of these, a pneumococcal serotype could be identified via the conventional Quellung reaction. Evidence of pneumococcal infection in 99 (84%) of 118 culture-negative PF samples was found on the basis of *ply* or *wzg* gene detection. PPE cases, diagnosed only by *ply* or *wzg* PCR, were significantly more likely to have received antimicrobial drug therapy before PF aspiration than patients with culture-positive pneumococcal PPE (92% vs. 53%; p<0.0001). Of the 99 culture-negative PF samples, 67 (65 *ply-*positive*/wzg*-positive and 2 *ply*-negative/*wzg*-positive) had a sufficient sample to enable serotype testing by PCR. In 52 of these samples, a serotype could be identified. Thus, a pneumococcal serotype was identified in 103 PF samples (Figure 2).

In addition, a predicted serotype based on MLST genotyping was established for 2 cases with negative PCR results and 6 cases for which neither conventional nor PCR-based serotyping was possible (Figure 2). Such predictions were possible because there is a strong relationship between serotype and MLST genotype for most genotypes (*16–18*; www.mlst.net), with the exception of a small number of well-known genotypes that are associated with different serotype variations. 

Eighty-one PF samples were fully genotyped, and 18 were partially genotyped (>4 alleles), by MLST. An ST was identified for 31 of the 99 culture-negative/*ply*-positive PPE. Among these 31 cases, there was full concordance between MLST data and PCR results for confirmation of predicted serotypes (Figure 2). Eighteen PF samples were partially genotyped by MLST: 2 were presumptive serotype 1 pneumococci based on 5–6 loci matching ST228; 1 was a presumptive serotype 5 based on 5 loci matching ST1223; 7 were genotyped at >4 loci and serotyped by PCR (serotype 1, n = 5; serotype 7F and 19A, n = 1 each); and 8 samples were partially genotyped at >4 loci (indicating presence of a pneumococcus), but PCR serotyping was either negative or not performed. Samples with predicted serotypes based on incomplete genotyping data were not included in further analyses.

### Serotype Distribution

Ten serotypes were identified among the 111 PPE cases with tentatively assigned or confirmed serotyping information (Table 2). Non–PCV7 serotypes caused 96 (89%) cases of PPE, including serotype 1, which was detected in 48% of the patient samples. Although a significantly higher proportion of PPE was caused by 7F in Seville and Malaga than in Barcelona, the contribution of other serotypes by region was not significantly different (Table 2). PCV7 uptake among PPE patients was significantly higher in Seville and Malaga than Barcelona (40% vs. 22%, p = 0.005), but there were no significant regional differences in vaccination status among children infected with serotype 1 (28% vs. 22%, p = 0.63).

**Table 2:** Pneumococcal serotypes identified among pleural fluid samples

Serotype*	Barcelona, no. (%), n = 56	Seville/Malaga, no. (%), n = 55	Total no. (%), n = 111	p value
1	27 (48)	26 (47)	53 (48)	0.92
7F	3 (5)	11 (20)	14 (13)	**0.02**
3	5 (9)	7 (13)	12 (11)	0.56
5	6 (11)	3 (5)	9 (8)	0.28
14	4 (7)	5 (9)	9 (8)	0.74
19A	6 (11)	2 (4)	8 (7)	0.27
9V	2 (4)	0	2 (2)	0.50
6A	2 (4)	0	2 (2)	0.50
8	0	1 (2)	1 (0.9)	1
19F	1 (2)	0	1 (0.9)	1

*7-valent pneumococcal conjugate vaccine serotypes include 4, 6B, 9V, 14, 18C, 19F, 23F. Pleural fluid samples were collected in Barcelona from October 1, 2003, through June 30, 2006, and in Seville and Malaga from January 1, 2005, through June 20, 2006. Serotypes were determined by Quellung reaction or PCR testing or predicted based on complete multilocus sequence typing, as described in the text. **Boldface** represents a statistically significant result.

Eight (15%) of 53 cultured pneumococci were intermediately penicillin resistant and 4 (8%) were resistant at high levels. Serotype 1, 3, 5, and 7F pneumococci were uniformly susceptible to penicillin and significantly more common among culture-negative than culture-positive PF samples (89% vs. 71%, p = 0.02)*.*

### Genotyping by MLST

Eighty-one PF samples were fully genotyped; 26 STs were identified (Table 3). Three of the major serotype 1 STs (*18*), ST228, ST304 and ST306, were identified, although ST228 was only detected in Seville and Malaga, and ST304 only in Barcelona (Table 3). Serotypes 5 and 7F were represented by globally distributed genotypes ST289 (Colombia^5^-19) and a closely related single-locus variant, ST1223; and ST191 (Netherlands^7F^-39), respectively.

**Table 3:** Sequence types and serotypes among 81 pneumococci detected in pleural fluid

Serotype*	Total	Sequence type (n)
1	43	306_and SLVs_ (23)† , 228 (11)‡, 304_and SLVs_ (8)†§, 2373 (1)
5	9	289_and SLV_ (9)†
3	8	180 (5), 260 (2), 2590 (1)§
14	7	156 (6), 17 (1)§
19A	6	276 (2), 81 (1)§, 202 (1)§, 1201 (1)‡, 2013 (1)§
7F	4	191 (4)
6A	2	135 (1)‡, 2377 (1)§
9V	2	838 (2)§

*Included are 8 strains that were culture and PCR negative, or not serotyped, but whose full genotyping by multilocus sequence typing (www.mlst.net) enabled serotypes 1, 5, and 7F to be predicted. These serotypes were predicted because in each case the sequence type (ST) was identical or closely related to a known genotype for serotypes 1, 5 or 7F that have never been identified with anything other than those respective serotypes. These included ST (no.): 306 (2), 228 (1), 2373 (1), 2378 (1), 2561 (1), 1223 (1), and 191 (1). †SLV, single locus variant (i.e., differs at only 1 MLST locus and thus is a closely related genotype). Major serotypes 1 and 5 ST groups included (no. strains): ST306 (18) and SLVs 2375, 2376, 2378, and 2561 (1 each); ST304 (5) and SLVs 2374 (2), 2371 (1); ST289 (5), and SLV 1223 (4). ‡Recovered only in Sevilla/Malaga. §Recovered only in Barcelona.

Six of 7 serotype 14–positive PF samples were ST156 (Spain^9V^-3). Genotypic diversity among the serotypes in this study was greatest for serotype 19A; 5 unrelated STs were detected, including ST81 (Spain^23F^-1). Such variants of ST81 have also previously been detected.

### IPD and Nasopharyngeal Carriage in Seville and Malaga

During 2001–2006, 180 cases of IPD involving children <14 years of age were diagnosed with IPD at Seville and Malaga hospitals, and 126 isolates were available for serotyping; 110 of these isolates were also genotyped. Twenty-three percent (29/126) of all IPD was due to serotype 1. Over this period, there was a statistically nonsignificant increase in the proportion of IPD cases due to serotype 1: 17% (2001–2003) vs. 27% (2004–2006), p = 0.19.

Twenty-four serotypes were identified; 10 serotypes caused both PPE and other IPDs (Table 4), and 14 serotypes caused only other IPDs (6B, 11, 13, 15A, 16, 18C, 22, 23A, 23B, 23F, 24, 33, 34, and 38). Serotype 1 isolates were almost exclusively associated with pulmonary disease, including bacteremic pneumonia (12/29, 41%) and PPE (15/29, 52%). The 3 major serotype 1 PPE genotypes were also found among this collection of serotype 1 IPD isolates, although ST304 was no longer detected after 2002 and ST306 was first detected in 2003. A retrospective analysis of serotype 1 invasive isolates submitted to the Spanish National Reference Laboratory since 1990 showed ongoing circulation of ST228 and ST304, but ST306 was only detected once before 2000 (1998; unpub. data).

**Table 4:** Contribution of PPE-associated serotypes and STs to IPD, Seville and Malaga, 2001–2006, and nasopharyngeal carriage in children <6 years of age, Seville*†

Serotype	No. (%) patients with IPD, n = 126	STs detected: diseases detected (no. patients), n = 111	Carriage, no. (%) patients, n = 194	STs detected in carriage (no. patients)	OR (95% CI)
1	29 (23)	**228**: P (7), PPE (6), A (1), B (1)	1 (1)	**306** (1)	**57.7 (7.7–429.9)**
		**306**‡ : PPE (7), P (3)			
		**304**§: PPE (1)			
14	22 (17)	**156**: P (7), PPE (4), M (2)	15 (8)	**156** (12)	**2.5 (1.3–5.1)**
		9: PPE (1)		409 (1)	
		62: P (1)		1684 (1)	
		124: PPE (1)		2607 (1)	
		2204: M (1)			
7F	10 (8)	**191**: PPE (4), P (3), M (1), B (1)	3 (2)	**191** (3)	**5.5 (1.5–20.3)**
19A	10 (8)	**276**: PPE (2), M (2), P (1)	13 (7)	**276** (2)	1.2 (0.5–2.8)
		**202**: B (1)		**202** (3)	
		**1201**: PPE (1)		**1201** (1)	
				199 (2)	
				433 (2)	
				392 (1)	
				2109 (1)	
				2609 (1)	
3	6 (5)	**260**: PPE (1), P (1), M (1)	7 (4)	**180** (3)	1.6 (0.5–4.6)
		**180**: PPE (1)		**260** (2)	
				2200 (2)	
6A	5 (4)	1150: M (1)	24 (12)	338 (8)	**0.29 (0.1–0.79)**
		1668: S (1)		386 (5)	
		1876: M (1)		1876 (3)	
				224 (2)	
				327 (1)	
				392 (1)	
				448 (1)	
				473 (1)	
				2201 (1)	
				2611 (1)	
5	3 (2)	**289**: PPE (2)	2 (1)	**289** (1)	2.3 (0.39–14.2)
		**1223**: P (1)		1540 (1)	
19F	3 (2)	87: C (1), B (1)	8 (4)	**81** (3)	0.57 (0.15–2.2)
		88: M (1)		87 (1)	
				179 (2)	
				63 (1)	
				2615 (1)	
9V	1 (1)	**838**: B (1)	3 (2)	**838** (3)	0.5 (0.05–5)
8	1 (1)	53: M (1)	0		–

*Culture-positive isolates only for IPD. **Boldface** indicates a statistically significant result and represents STs that were identified among pleural fluids during the study period in Seville, Malaga, and Barcelona. An OR demonstrating the potential for each serotype to cause invasive disease, relative to its prevalence in nasopharyngeal carriage, was also calculated (*16*). Serotypes 6A, 19F, and 9V were associated with PPE in Barcelona (but not Seville and Malaga) and are included here for a complete list of invasive serotypes with any association to PPE among the 3 locations; however, data presented here are only from Seville and Malaga. Serotypes identified in IPD cases but not among children with PPE: 6B, 11, 13, 15A, 16, 18C, 22, 23A, 23B, 23F, 24, 33, 34, 38. †PPE, pediatric parapneumonic empyema; IPD, invasive pneumococcal disease; ST, sequence type; OR, odds ratio; CI, confidence interval; A, arthritis; B, occult bacteremia; P, pneumonia; M, meningitis; S, sepsis; C, orbital cellulitis. ‡First detected in 2003. §First detected in 2002.

Serotype 14 was the second most common IPD-causing serotype, with an overall prevalence of 17% (23% in 2001–2003 and 12% in 2004–2006; p = 0.12). The major serotype 14 genotype (ST156) identified in PF samples was also detected throughout the entire 2001–2006 period among carriage isolates and in culture-positive IPD cases, mainly causing pulmonary disease (Table 4). Ten (8%) cases of culture-positive IPD were due to serotype 7F, 9 of which were detected after 2004. ST191 was the only serotype 7F genotype in IPD and NP carriage.

### Serotype-Specific Differences in Clinical Epidemiology, Inflammatory Markers, and Outcome

PPE-associated serotypes were divided into 3 groups: 1) serotypes 1, 5, 7F, and 14, consistently associated with the highest estimates of serotype-specific high invasive disease potential (HIDP) (*16*,*17*,*19*); 2) serotype 3 alone; and 3) serotypes 6A, 9V, 19A, and 23F, which have a low invasive disease potential (LIDP) (*16*,*17*,*19*). Odds ratio estimates of invasive disease potential demonstrate as much as 60- to 120-fold variation between the most invasive (1, 4, 5, 7, 14, 18C) and the least invasive (3, 6A, 15, 23F) serotypes/serogroups (*16*,*19*).

PPE cases with HIDP serotypes were older than those with LIDP serotypes (median ages 56 and 24 months, respectively; p = 0.0001) (Table 5). Among HIDP PPE cases, 74% were due to serotypes 1 (n = 53) and 5 (n = 9) and comprised children >36 months of age, whereas serotype 14 (n = 9) only caused PPE in patients <36 months of age (data not shown; p = 0.0001). Serotype 3 PPE was associated with significantly more complications than PPE caused by HIDP and LIDP serotypes combined (p = 0.004). No other characteristics differed significantly between individual groups (Table 5).

**Table 5:** Characteristics of children hospitalized with PPE, by serotype category, excluding patients with serious underlying disease  (n = 3)*

Characteristic	HIDP serotypes, n = 84	Serotype 3, n = 11	LIDP serotypes, n = 13	p value
Median age, mo (range)	55.6 (2–180)	37.9 (9–71)	24 (2–36)	0.0001†
Median hospital stay,‡ d (range)	13 (4–38)	15 (9–29)	10 (6–24)	0.042§
Complications, % patients¶	10	45	0	0.004#

*PPE, pediatric parapneumic empyema; HIDP, high invasive disease potential; LIDP, low invasive disease potential. HIDP serotypes: 1, 5, 7F, and 14; LIDP serotypes: 6A, 9V, 19A, and 19F (*16*,*17*,*19*). All results shown were statistically significant (p<0.05). There were no significant differences between groups for the following variables: median days febrile preadmission; preadmission antimicrobial therapy; intensive care unit admission; mean leukocyte count; mean C-reactive protein; mean pleural fluid glucose; mean pleural fluid pH; mean lactate dehydrogenase; median days to thorachocentesis; referral; primary fibrinolytics or thoracoscopy; or oxygen requirement >4 d. †HIDP was compared with LIDP by post hoc analysis. ‡Since being admitted to first center. §No significant differences between individual groups by post hoc analysis (p = 0.023 for comparison between serotype 3 and LIDP) ¶Complications included (no. patients): bronchopleural fistula (3), pyopneumothorax (2), pneumatoceles (4), lung abscess (1), mechanical ventilation  >48 h (2), severe anemia requiring blood transfusion (2), severe hypoalbuminemia requiring seroalbumin replacement (1). #Serotype 3 compared with HIDP and LIDP groups combined.

## Discussion

In this study, we used molecular techniques to sensitively evaluate PPE epidemiology among a large number of patients in geographically diverse locations of Spain. There was evidence of pneumococcal infection in most of the culture-positive and culture-negative cases of PPE, which was mainly associated with nonvaccine serotype 1 followed by 3, 5, 7F, and 19A, as well as vaccine serotype 14. Serotypes 1, 3, and 14 in particular are well-known PPE-associated serotypes (*2*,*4*,*7*,*20*,*21*). Antimicrobial drug–susceptible serotypes 1, 3, 5, and 7F were overrepresented in culture-negative PF samples, pointing to an important potential bias in PPE surveillance when surveillance is based solely on conventional microbiologic culture methods. Infection with serotype 3 was a risk factor independently associated with PPE complications, a finding also seen in a US study (*22*).

Serotype 1 has also been the most prevalent IPD serotype among Spanish children <14 years of age, representing 5%, 11%, and 27% of all culture-positive pediatric IPD isolates sent to the Pneumococcal Reference Laboratory in 1997, 2003, and 2006, respectively (*23*). However, the increase in serotype 1 disease cannot easily be explained by a vaccine effect, in part because PCV7 coverage was relatively low in both regions for much of the study period. Registered in Spain in June 2001, PCV7 had a low initial uptake that increased to a reported coverage of 34%–45% in 2004–2005 (*24*,*25*).

In addition, increased PPE incidence largely caused by serotype 1 was reported in the United States and the United Kingdom in the decades before PCV7 introduction in 2000 and late 2006, respectively (*4*,*6*,*20*). Previous studies have suggested that the high year-to-year variability of serotype 1 and 5 disease may represent large-scale outbreaks of a cyclical nature (*26*–*28*).

However, the observation in this study that 2 of the 3 MLST genotypes of serotype 1 (ST228 and ST304) had been “resident” in Spain at least since 1990 indicates that serotype 1 PPE increases in Spain were likely not due to a recent introduction of a specific clone. In general, MLST analyses demonstrated that the recent increase in PPE was mainly due to pneumococcal STs previously described to be present in Spain and other European countries for some years (*18*,*27*,*29*–*31*).

Our study has several limitations. First, the limited study period did not enable a longer-term analysis of PPE epidemiology. Second, our analyses relied exclusively on serotype identification and MLST genotyping, neither of which detects differences in virulence factors apart from the serotype. Genetic factors independent of the capsule have been associated with invasiveness and disease severity (*17*,*32*,*33*). Third, other factors that may also modulate the epidemiology of PPE (e.g., differences in case ascertainment between the retrospective and prospective studies, viral infections, or climatic patterns [*34*]) were not evaluated. Fourth, it remains difficult to evaluate the PCV7 impact because reliable written immunization registries detailing the number of administered doses are lacking, and thus vaccine coverage figures mainly come from parent reporting. Finally, the results obtained here may not apply to less severe pneumonia cases, whose etiology may be qualitatively different.

Unfortunately, PCV7 has a serotype coverage of only 11%–14% (including the cross-reactive 6A) in the population of PPE patients. However, conjugate vaccines containing serotypes 1, 5, and 7F, such as the newly developed 10-valent pneumococcal *Haemophilus influenzae* protein D conjugate vaccine candidate (*35*), could increase the serotype coverage for PPE up to 80%; the subsequent addition of serotypes 3 and 19A in vaccine candidates currently in development would add an additional 18% of coverage (*35*). Finally, continued epidemiologic surveillance with molecular diagnostic techniques will be crucial to understanding this serious pediatric disease.
